# Incidence of breast cancer in Italy: mastectomies and quadrantectomies performed between 2000 and 2005

**DOI:** 10.1186/1756-9966-28-86

**Published:** 2009-06-19

**Authors:** Prisco Piscitelli, Antonio Santoriello, Franco M Buonaguro, Massimo Di Maio, Giovanni Iolascon, Francesca Gimigliano, Alessandra Marinelli, Alessandro Distante, Giuseppe Serravezza, Emiliano Sordi, Katia Cagossi, Fabrizio Artioli, Michele Santangelo, Alfredo Fucito, Raffaele Gimigliano, Maria Luisa Brandi, Massimo Crespi, Antonio Giordano

**Affiliations:** 1 CROM Cancer Research Center, Mercogliano, Italy; 2 Institute of Internal Medicine, University of Florence, Florence, Italy; 3 Institute of Rehabilitative Medicine, Second University of Naples, Naples, Italy; 4 National Cancer Institute IRCCS Pascale, Naples, Italy; 5 Local Health Authority of Naples (ASL NA1), Italy; 6 ISBEM Research Institute, University of Pisa, Pisa, Italy; 7 L.I.L.T.-ILMA Research Center, Lecce, Italy; 8 Institute of Clinical Oncology, Carpi Hospital, Modena, Italy; 9 Sbarro Health Research Organization, Philadelphia, USA; 10 Department of Public Health, National Cancer Institute Regina Elena, Rome, Italy; 11 Department of Human Pathology & Oncology, University of Siena, Siena, Italy

## Abstract

**Objectives:**

We aimed to determine the incidence of women's breast cancer in Italy without using statistical approximations.

**Methods:**

We analyzed the national hospitalizations database at the Ministry of Health to calculate the number of major surgeries in Italian women (mastectomies and quadrantectomies) due to breast cancer between 2000 and 2005, overall and by age groups (<44, 45–64, 65–74 and ≥ 75 years old).

**Results:**

Over the six years examined, an overall number of 100,745 mastectomies and 168,147 quadrantectomies were performed. A total of 41,608 major surgeries due to breast cancer were performed in the year 2000 and this number rose to 47,200 in 2005, with a 13.4% increase over six years.

**Conclusion:**

by analyzing the hospitalizations database concerning major breast surgery, incidence of breast cancer in Italy was found to be 26.5% higher than the official estimations which have been computed using statistical models (namely 47,200 vs. 37,300 cases in year 2005).

## Introduction

Women in Italy account for 30 out of 59 million inhabitants, thus representing more than 50% of the entire population [1]. According to the Italian National Institute for Statistics (ISTAT), women's life expectancy at birth increased by a rate of 4 months per year from 1950 to 2002, reaching 86.6 years. This value is estimated to rise up to 87.4 years by 2010 [1]. After cardiovascular diseases, tumors represent the first cause of death among women in Italy, each year killing 119 and 38 per 10,000 women in the 55–74 and ≥ 75 age groups, respectively [2,3]. Breast cancer is the leading tumor among women in Italy [1]. The risk of developing breast cancer is related to a number of factors including the events of reproductive life and lifestyle factors that modify endogenous levels of sex hormones [4]. Diet has been also found to play an important role in the etiology of breast cancer [5]. Official data from the Italian Ministry of Health have estimated the total breast cancer incidence at 37,300 new cases in year 2005, with an overall prevalence of 416,000 cases (women living with the cancer) [6]. The incidence per age group was estimated to exceed 100 new cases every 100,000 women ≥ 40 years of age, rising up to 200 new cases and over 300 cases in the ≥ 50 and ≥ 60 year-old groups, respectively [2,7]. The number of deaths due to breast cancer in the Italian female population represented about 18% of the total cancer mortality rate in 1998, but the mortality rate has been reduced by 20% in the last 10 years [2,7]. In the year 2008 a total of 11,000 deaths were attributable to breast cancer among Italian women [2].

Until now, official epidemiological data concerning the incidence of breast cancer in Italy have been computed by using a statistical model (MIAMOD, Mortality-Incidence Analysis MODel), which represents a back-calculation approach to estimate and project the morbidity of chronic irreversible diseases, starting with mortality and survival data [6,8,9]. This kind of approach is justified in light of the need to evaluate the incidence of all tumors, but may underestimate the incidence of breast cancers, since many of the deaths occurring at home or in hospital settings could be attributed to cardiovascular causes on the statistical forms filled out by physicians. The availability of accurate incidence data concerning breast cancer is of particular relevance, due to the need to evaluate the progress achieved through preventive screening campaigns. In this study, we aimed to determine the incidence of breast cancer in Italian women, by using an alternative methodology that avoids statistical models, in order to obtain data which could more closely reflect reality, through the analysis of performed surgical procedures. This methodological approach has never been used in analyzing cancer incidence; however it has already been validated in studies carried out in Italy [10-17], Germany [18] and France [19] concerning other surgical procedures, which aimed to evaluate incidence of osteoporotic fractures, myocardial infarctions and heart failure.

## Materials and methods

Information concerning all hospitalizations occurring in Italian public and private care setting are registered in hospital discharge records, which are collected at the Italian Ministry of Health (national hospitalization database, SDO). These information are anonymous and include patient's age, diagnosis, procedures performed, and the length of the hospitalization. Thanks to the availability of this huge database, we hypothesized to overcome limitations of the MIAMOD model in estimating the burden of breast cancer. Therefore, we analyzed the national hospitalization database (SDO) maintained at the Italian Ministry of Health between 2000 and 2005 (the latest year available for consultation) searching for mastectomies and quadrantectomies, the main surgical procedures performed in case of breast cancer. We assumed that the number of these procedures closely reflected the number of new breast cancers (namely the incidence) as it is mandatory a very short time between tumor diagnosis and surgery (no more than 30 days) [20,21].

The assumptions concerning the weakness of the MIAMOD model in evaluating breast cancer burden and the possibility to better estimate the real incidence by computing the number of surgical procedures have been accepted by a panel of expert epidemiologists, surgeons, oncologists and radiologists (co-authors of this article) before starting the study. We have reported all cases of women who underwent major surgery (mastectomies and quadrantectomies) due to breast cancer. Therefore, it is possible that we computed twice some patients who underwent two operations in the same year, and there is the possibility of having considered some new incidental cases diagnosed in the year preceding the time of the operation (i.e. during the month of December). However, this effect was considered to be minimized because of the short time elapsing between diagnosis of breast cancer and surgery [20,21], and when looking at the overall number of surgical interventions performed over the whole period considered (2000–2005), which actually includes all the new cases diagnosed across the 6 examined years. Furthermore, the possibility of having computed the same patient two times (major surgical procedures performed twice on the same person) is a very uncommon occurrence in our clinical experience, based on a 1.000 patients clinical setting who underwent breast surgery at Second University Hospital of Naples.

In the analysis of the national hospitalization database (SDO), "mastectomy" and "quadrantectomy" were defined respectively by intervention codes 85.4 (all the extensions) and 85.22. We considered both ordinary hospitalization regimen and day hospital. Tumorectomies, which represent the elective surgical treatment for minimal lesions (i.e. in situ carcinoma) have been excluded from this study because a specific code for this procedure does not exist. However, minimal invasive cancers, which do not need further surgical treatments other than biopsy, represent only a small percentage (approximately below 5%) of the overall excision biopsies (intervention code 85.21).

Data were stratified into four age groups (25–44, 45–64, 65–74 and ≥ 75 years) and were processed using Stata (StataCorp, College Station, USA) and Excel (Microsoft, Redmond, USA) softwares. We performed descriptive statistical analyses of the incidence in each age subgroup across the six examined years. The study period (from year 2000 to 2005) was chosen because it reflects the most recently available nationwide clinical (hospitalization records) and demographic data. Population data were obtained from the National Institute for Statistics (ISTAT) for each of the considered years [1].

## Results

A total of 100,745 mastectomies and 168,147 quadrantectomies were performed over six years, resulting in a total of 268,892 major surgical procedures (Table 1). The overall number of surgeries (mastectomies + quadrantectomies) due to breast cancer was 41,608 in the year 2000, 43,443 in 2001, 44,491 in 2002, 45,065 in 2003, 47,085 in 2004, and rose up to 47,200 operations in year 2005, with a 13.4% increase over six years (Table 1, Table 2, Table 3). If compared to the official data of the Italian Ministry of Health, which are based on the MIAMOD model approximations, there is a difference of about 26.5% regarding the incidence of breast cancers in the year 2005 (37,300 vs. 47,200 new cases, respectively). Considering all the six years together, the majority of surgical procedures due to breast cancer were performed in patients between 45 and 64 years of age (55%; n = 124,241 operations).

**Table 1:** Total number of major surgical interventions (mastectomies and quadrantectomies) performed in Italy between 2000 and 2005 (SDO Italian hospitalizations database)

**Age group**	***2000***	***2001***	***2002***	***2003***	***2004***	***2005***	***Six years total***
*25–44*	**5 291**	**5 694**	**5 854**	**6 063**	**6 674**	**6 808**	**36 384**

*45–64*	**19 485**	**20 438**	**21 130**	**20 748**	**21 142**	**21 298**	**124 241**

*65–74*	**9 671**	**9 966**	**10 356**	**10 145**	**11 209**	**10 808**	**62 155**

*> 75*	**7 161**	**7 345**	**7 151**	**8 109**	**8 060**	**8 286**	**46 112**

*Sub total*	**41 608**	**43 443**	**44 491**	**45 065**	**47 085**	**47 200**	**268 892**

**Table 2:** Mastectomies performed in Italy between 2000 and 2005 (SDO Italian hospitalizations database)

**Age group**	***2000***	***2001***	***2002***	***2003***	***2004***	***2005***
25–44	**1 853**	**1 980**	**1 914**	**2 031**	**2 064**	**2 000**

% increase vs. prev. year	*-*	*+6.85%*	*-3.33%*	*+6.11%*	*+1.62%*	*-3.10%*

45–64	**6 705**	**6 677**	**6 776**	**6 197**	**6 029**	**5 780**

% increase vs. prev. year	*-*	*-0.41%*	*+1.14%*	*-8.54%*	*-2.71%*	*-4.13%*

65–74	**4 228**	**4 160**	**4 159**	**3 831**	**3 786**	**3 828**

% increase vs. prev. year	-	-6.05%	-0.02%	-7.88%	-1.17%	+1.10%

> 75	**4 497**	**4 464**	**4 604**	**4 607**	**4 326**	**4 249**

% increase vs. prev. year	**-**	-0.73%	+3.13%	+0.06%	-6.09%	-1.77%

Total	**17 283**	**17 281**	**17 453**	**16 666**	**16 205**	**15 857**

% increase vs. prev. year	-	-0.01%	+0.99%	-4.50%	-2.76%	-2.14%

**Table 3:** Quadrantectomies performed in Italy between 2000 and 2005 (SDO Italian hospitalizations database)

**Age group**	***2000***	***2001***	***2002***	***2003***	***2004***	***2005***
25–44	**3 438**	**3 714**	**3 940**	**4 032**	**4 610**	**4 808**

% increase vs. prev. year	*-*	*+8.02%*	*+6.08%*	*+2.33%*	*+14.33%*	*+4.29%*

45–64	**12 780**	**13 761**	**14 354**	**14 551**	**15 113**	**15 518**

% increase vs. prev. year	*-*	*+7.67%*	*+4.30%*	*+1.37%*	*+3.86%*	*+2.67%*

65–74	**5 443**	**5 806**	**6 197**	**6 314**	**7 423**	**6 980**

% increase vs. prev. year	*-*	*+6.66%*	*+6.73%*	*+1.88%*	*+17.56%*	*-5.96%*

> 75	**2 664**	**2 881**	**2 547**	**3 502**	**3 734**	**4 037**

% increase vs. prev. year	*-*	*+8.14%*	*-11.59%*	*+37.49%*	*+6.62%*	*+8.11%*

Total	**24 325**	**26 162**	**27 038**	**28 399**	**30 880**	**31 343**

% increase vs. prev. year	*-*	*+7.55%*	*+3.34%*	*+5.03%*	*+8.73%*	*+1.49%*

The total number of mastectomies went from 17,283 in the year 2000 to 15,857 in 2005 (with a reduction of about -8.2% across the six examined years). We observed in most age groups (45–64, 65–74 and ≥ 75 years) a reduction in the number of mastectomies between year 2005 vs. year 2000, with the only exception of women aged <45 years old (an age group excluded from national screening campaigns), where an increase of 7.9% in the number of mastectomies was found (Table 2). This finding could be related to a late diagnosis of breast tumors in women aged 25–44, thus requiring disruptive surgery. On the other hand, there was an increase of 28.8% in the overall number of quadrantectomies, passing from 24,325 (year 2000) to 31,343 in 2005. The increase of quadrantectomies was shown in all the four age groups (Table 3). Even in the youngest age group, quadrantectomies increased more than mastectomies, as a 28.6% increase (+1517 cases) in the overall number of procedures (mainly quadrantectomies) was found in women <45 years of age, and accounted for about 15% of the overall increase observed across the six examined years in the total number of surgeries.

A total of 38,164 mastectomies and 86,077 quadrantectomies were performed in patients aged between 45 and 64 years across the six years examined, with quadrantectomies increasing by a rate of about 21.0%. Similarly, in patients aged 65–74 and ≥ 75 years old, we observed an increase of 28.3% (+1537 cases) and 51.5% (+1373 cases) respectively, concerning the number of quadrantectomies performed between 2000 and 2005. In table 2 and table 3 we have also shown the percentage of average yearly increase, and the % increase vs. previous year per each age group. According to our data concerning major breast surgeries, the overall incidence of breast cancer per 100.000 women aged 0–84 years old was 141.80 in year 2000 and 160.85 in 2005, with a 13.4% increase (Table 4). The incidence rate per 100.000 women aged 0–84 years old found in our study is 72% higher than that provided by official estimations of the Ministry of Health (93.0 per 100,000 women aged 0–84 years) based on the MIAMOD model for the same year 2005 [6]. According to our data, in women aged ≥ 75 years old, incidence of breast cancer per 100.000 was 208.4 in year 2000 and 241.2 in 2005, with an increase of 15.7% across six years. Between 2000 and 2005, the increase in the incidence of breast cancer per 100.000 women was +11.7%, +9.3%, and +28.6 in women aged 65–74, 45–64, and 25–44 respectively (Table 4). The highest increase in the incidence rate per 100.000 women was observed in this latter age group (<45 years old), and it is of special interest because it has been found in a younger population which is not taking part into screening campaigns at the present.

**Table 4:** Age standardized incidence of breast cancer per 100.000 women (Italy 2000–2005)

***Age group***	***2000***	***2001***	***2002***	***2003***	***2004***	***2005***	***2005 vs. 2000 increase***
25–44 years old	59.58	64.12	65.92	68.28	75.16	76.67	***+28.68%***

45–64 years old	256.91	269.47	280.97	273.56	278.75	280.81	***+9.30%***

65–74 years old	289.97	298.81	310.51	304.18	336.08	324.06	***+11.75%***

≥ 75 years old	208.45	213.81	208.16	235.95	234.62	241.20	***15.71%***

Overall incidence 0–84 years old	141.80	148.05	151.61	153.58	160.46	160.86	***13.44%***

## Discussion

The direct analysis of the national hospitalization database (SDO) allowed us to overcome the limitations related to the use of statistical models, and particularly those of the official reports based on model approximations (i.e. the MIAMOD model). By analyzing hospitalization database concerning major breast surgery, the incidence of breast cancer in Italy was found to be 26.5% higher than the official incidence estimated in year 2005 (the last year examined) by the Italian Ministry of Health. A full-evaluation of breast cancer incidence would have required the analysis of tumorectomies. Therefore, our results should be regarded as conservative.

The improvement of women's compliance to the screening campaigns could have contributed to reducing the number of mastectomies across the six examined years as a result of earlier detection of malignancies. Similarly, the adoption of proper screening campaigns could have increased the overall number of surgical procedures due to breast cancer, as a consequence of a higher number of new diagnoses [22]. It must be pointed out that one of the major increases (+ 28.6%) in the number of surgeries (mainly quadrantectomies) has been observed in women aged <45 years old., and that we have found an increase in the number of mastectomies only in this younger age group, possibly as a consequence of delayed diagnoses. In the same young age group, it has been observed the highest incidence rate of breast cancer per 100.000 women, thus suggesting the need for an effective screening campaign even before the age of 45 years. However, the number of quadrantectomies remarkably increased also in women aged 45–64, 65–74 and mostly in those >75 years, which actually represent the female population currently invited to screening campaigns, according to the most recent nationwide studies [23,24]. The 6% reduction observed between 2004 and 2005 in the number of quadrantectomies performed in women aged 65–74 years (which went from 7,423 to 6,980) should not be regarded as significant because in the previous two years (2003 vs. 2004) we had found the biggest increase (+17.6%; corresponding to 1109 cases) observed in this age group, with quadrantectomies passing from 6,314 (year 2003) to 7,423 (year 2004) within only one year.

This study points out the limitations of statistical models in providing firm data about the incidence of malignancies, because these models are based on ISTAT mortality rates. Acute mortality rate of breast cancer is supposed to be around 5% [2,7], while mid-term (1-year) mortality is estimated to be between 20 and 25% [2,7]. There is the possibility that a percentage of women who died in hospital or at home as a consequence of breast cancer could be assigned to another "final" cause of death (i.e., respiratory or cardiac arrest) rather than to breast cancer. Given the continuously increasing trend of breast cancer incidence and costs, effective preventive strategies should also include actions aimed to remove the primary causes of these malignancies, such as environment pollution due to dioxins and other carcinogens.

## Conclusion

This study shows that, in the Italian female population, the number of surgical procedures due to breast cancer has grown across the six examined years, especially in women aged less than 45 and over 75 years old, exceeding 47,000 new cases in 2005. Breast cancer incidence in Italy, when evaluated on hospital database, was 26.5% higher than the official data provided by the Italian Ministry of Health (47,200 vs. 37,300 new cases, respectively), which are based on MIAMOD model approximations (Mortality-Incidence Analysis MODel). This study confirms that the use of the national hospitalization database is useful for estimating breast cancer incidence, even though further researches should also deeply investigate the burden of tumorectomies and evaluate inter-regional differences, which were not considered in this analysis.

## Competing interests

The authors declare that they have no competing interests.

## Authors' contributions

PP, AS, FMB, MDM, AG conceived of the study, and participated in its design and coordination; GI, FG, AM, AD, MLB, MC, AG participated in the design of the study; GS, ES, FA, MS, AF carried out the clinical re-evaluation of the study results. All authors have read and approved the final manuscript.
